# Assessment of meridic larval and adult diets for mass rearing of *Bactrocera dorsalis* (Diptera: Tephritidae)

**DOI:** 10.1371/journal.pone.0335213

**Published:** 2025-10-27

**Authors:** Mahfuza Momen, Md. Shahjalal, Md. Ashikur Rahman, Md. Aftab Hossain, Md. Kamruzzaman Munshi, Kajla Seheli

**Affiliations:** 1 Insect Biotechnology Division, Institute of Food and Radiation Biology, Atomic Energy Research Establishment, Bangladesh Atomic Energy Commission, Dhaka, Bangladesh; 2 Food Safety and Quality Analysis Division, Institute of Food and Radiation Biology, Atomic Energy Research Establishment, Bangladesh Atomic Energy Commission, Dhaka, Bangladesh; National Institute of Agricultural Research - INRA, MOROCCO

## Abstract

The oriental fruit fly, *Bactrocera dorsalis* (Diptera: Tephritidae), is a widespread pest in Bangladesh. Sterile Insect Technique (SIT) offers a solution for effectively suppressing this fruit fly species. However, SIT involves mass rearing of fruit fly species in a laboratory where a standardized artificial rearing diet is crucial for ensuring uniform growth, development, and reproduction. In this study, we assessed efficacy of a new formulated gel-based meridic larval diet as well as protein and carbohydrate rich adult diets for the rearing of *B. dorsalis* in laboratory conditions. Proximate analysis was conducted for our formulated rearing diets to determine the content of moisture, protein, fat, carbohydrate, and ash. For our formulated diets, several key biological parameters, including egg hatching rate, pupation rate, pupal weight, adult emergence, adult growth, sex ratio, and flight capacity, were assessed. Statistical analysis using Tukey box plots revealed a significant improvement for the laboratory reared body parameters of adults while maintained in meridic diets, as compared to their wild counterparts. Adults fruit flies reared on our formulated meridic adult diets exhibited sufficient longevity, especially when compared to those provided with only water. In addition, our study presents survival analysis using non-parametric Kaplan–Meier estimator and Weibull parametric model. Our findings indicate that the formulated diets presented in this study can be effectively incorporated into *B. dorsalis* laboratory mass-rearing, meeting the required standard quality parameters outlined in the FAO/IAEA/USDA mass-rearing guideline of tephritid fruit flies.

## Introduction

*Bactrocera dorsalis*, commonly known as oriental fruit fly, is a highly destructive dominant pest of fruits and vegetables in Bangladesh [1–3]. *B. dorsalis* females lay their eggs in the pulp of a wide range of hosts, leading to a significant crop damage as the larvae hatch and begin feeding on fruits. After progress through three stages of development, the larvae eventually leave the fruits to pupate in the soil from where adults emerge. Again, they will continue the cycle by laying eggs into new fruits, ultimately leading to further losses to farmers [4]. The implementation of proper control measures for this pest is crucial in order to mitigate economic loss and to ensure the protection of agricultural yields. Among various approaches of effective pest management strategies, the Sterile Insect Technique (SIT) offers an effective and sustainable approach for population control of *B. dorsalis* [3,5].

**Larval diet:** Mass rearing of *B. dorsalis* larvae under laboratory conditions is one of the crucial primary steps for a successful implementation of SIT. In laboratory rearing systems, a larval diet creates an environment for larvae to grow and feed before they pupate. By ensuring optimal nutrition and conditions for larval development, we can increase the production of sexually competitive adults for a successful SIT application. Therefore, the implementation of appropriate cost-effective larval diets that ensure optimal larval development and lead to mass production of healthy competitive males, is an integral part for successful SIT programs [6]. However, suitable larval and adult diets for mass rearing of this fruit fly in a laboratory can be an expensive and challenging process [7]. The primary reason for this is the absence of a standard rearing diet that can be formulated from locally available and affordable ingredients.

Primarily, whole host fruits like papaya, banana, guava etc. were used to establish the colony in many laboratories on a small scale [8]. However, fruits are typically seasonal, which limits the availability of fresh fruits in consistent quantities needed for a larval diet. Furthermore, use of natural hosts for laboratory rearing of fruit flies introduces inconsistencies and variability in quality [9]. Therefore, alternative larval diets are required to overcome these limitations.

A major advancement in the laboratory rearing tephritid fruit fly larvae was the incorporation of dehydrated plant materials (e.g., carrot powder) and dried yeast into their diets. The incorporation of dehydrated plant materials significantly improved larval rearing and resulted in higher pupal yields [10,11]. Over the years, several artificial larval diets have been developed, used, and evaluated for mass rearing of *B. dorsalis* [12–15]. Mass production of *B. dorsalis* is usually based on a diet that includes water, sugar and yeast as nutrients, and grain powder as a bulking agent. However, different types of bulking agents (e.g., corn cob powder, bagasse, wheat bran, etc.) have major drawbacks, such as excessive heat generation due to microbial activity and a tendency for mycotoxin contamination, negatively impacting growth and survival. Another significant drawback of this formulation is the substantial waste generation due to the use of bulking agents. This undesirable waste, which remains useless as well as negatively impacts the rearing environment, also increases production cost. Therefore, researchers are searching for a new diet formulation that would ensures sustainable mass rearing while reducing bulking agents.

Gelling agents can serve as an essential purpose in stabilizing dietary ingredients by preventing undesired chemical reactions among ingredients. These gelling agents are promising additions to larval diets, transforming high-water content mixtures into semi-solid gels while ensuring uniform ingredient distribution. For instance, agar has exceptional water retention properties, capable of holding a high percentage of water in its gel form [16]. This attribute of agar helps to reduce desiccation as well as maintain the larval diet texture [17]. As a result, gel-based diets emerged as a promising alternative for larval rearing. In our laboratory, a gel-based larval diet was adopted for rearing *B. dorsalis* [15]. However, for scaling up mass-production of *B. dorsalis* from small-scale laboratory rearing, there is a search for a more efficacious and cost-effective larval diet formulation. A very limited number of research works were reported on the larval gel diets [14,15,17–19]. Previous research by Khan et al. explored a gel-based larval diet to optimize the rearing of a local wild *B. dorsalis* population [12,15]. However, in the previous study, a limited set of biological quality parameters was examined without analyzing the nutrient contents of the diet. In addition to that various parameters have interdependent relationships with one another. Therefore, there is a need for more comprehensive studies where relevant parameters are studied together to get a more detailed understanding of the efficacy of diet formulation.

**Adult diet:** Healthy and vigorous fruit flies are essential for research and SIT programs. A proper diet for adult rearing is crucial for maintaining the quality of adult fruit flies. However, mass rearing of the adult stage presents subsequent challenges [20–23]. While newly emerged adults can survive on sugar and water, dietary protein is also essential for gonad maturation and male mating performance. Therefore, understanding the nutritional requirements and dietary preferences of adult Tephritidae is extremely important for developing effective management strategies as well as improving the quality of mass-reared individuals for pest control programs. Careful consideration of the sources and balance of key nutrients like proteins, carbohydrates, vitamins, and lipids is essential for formulating an efficient artificial diet for adult *B. dorsalis*. Previous studies have shown that diets containing yeast or yeast derivatives enhance egg production and overall fitness of various Tephritidae species [24,25]. A common formulation includes a mixture of sugar and yeast. This combination provides the essential nutrients for adult flies, promoting mating behavior and longevity [26]. Containing vitamins in the diet is also important for reproductive health and stress resistance as well as overall fitness of adult fruit flies. The composition of the adult diet directly affects sexual maturation rates, body weight, and mating success. Diets that are rich in vitamins and proteins can improve longevity under stressful conditions, which is critical for maintaining a healthy population during mass rearing.

An ideal diet should maintain a consistent composition that prevents fermentation as well as eliminates the necessity for additional supporting substrates and minimizes waste during the rearing process. Such a diet simplifies rearing procedures and ensures a consistent nutritional intake, leading to more standardized insect quality. Several formulations of artificial adult diet have been developed using low-cost ingredients for different tephritid species [20,25,27,28]. However, advancement in the diet formulations is still necessary to optimize it further, leading to improved stability and reduced fermentation, especially in the subtropical monsoon climate of Bangladesh.

Despite progress in adult diet development, limited information is available regarding the effect of both dry and liquid formulations of adult diets on the lifespan as well as overall fitness of adult oriental fruit flies. Further research that takes into account various parameters simultaneously is essential for understanding the long-term impacts of adult diets on the lifespan and quality of adult *B. dorsalis*. To assess the quality and longevity of *B. dorsalis* adults under laboratory rearing conditions, in this study we conducted a series of experiments using two types of adult diet formulations (dry and liquid diet).

In this work, we present a comprehensive assessment and comparison of the effectiveness of our new formulated gel-based meridic diet for larvae along with yeast-based diets for adult fruit flies. Our study presenting Tukey box plots demonstrated a significant improvement in adult body parameters and longevity while using the new formulated diets. In addition, we preformed survival analysis using non-parametric Kaplan–Meier estimator and Weibull parametric model. The findings in this study will contribute to the optimization of rearing techniques ensuring the sustainability and efficiency of the mass production of this fruit fly species.

## Materials and methods

This study was conducted at Insect Biotechnology Division laboratory of Institute of Food and Radiation Biology, Atomic Energy Research Establishment, Bangladesh. To ensure strong and reliable results, all experiments were replicated at least three times with sufficient sample sizes to minimize the influence of random sampling error. The fruit fly species, *B. dorsalis*, used in this experiment was obtained from a laboratory colony established from a local pest population. The insectary was maintained at 27 ± 1 °C, 70 ± 5% relative humidity (RH), with a 14-h light:10-h dark photoperiod. All the procedures used in this study are summarized below.

### Larval diet formulation

In this study, the new formulated larval diet used for rearing *B. dorsalis* larvae was gel-based. To prepare this larval diet, all the ingredients (sugar, preheated baking yeast, soy protein, soy bran, sodium benzoate, citric acid, agar and distilled water) were mixed well (see Table 1). The mixture was carefully heated until it reached a vigorous boil, and then the boiling temperature was maintained for 5 minutes to ensure thorough heating. Aliquots of 333 mL of the hot gel-based diet were then dispensed into individual larval rearing trays (14 cm × 20 cm) and left to solidify at room temperature for 2-3 hours.

**Table 1:** Formulation of gel-based larval diet for rearing *Bactrocera dorsalis.*

Ingredients	Source	Gel diet (per 1 L)
Agar	Sigma-Aldrich^®^	3.40 g
Sugar	Advanced Chemical Industries (ACI),Bangladesh	97.44 g
Baker’s yeast	Angel Yeast Co., Ltd. China	81.6 g
Soya protein	Standard Agro food Manufacturing (PVT.) Ltd., Bangladesh	40.8 g
Soya bran	Locally produced	40.8 g
Citric acid	Sigma-Aldrich^®^	15 g
Sodium benzoate	Sigma-Aldrich^®^	3.2 mL
Distilled water	Water distillation unit of laboratory	800 mL

### Adult diet formulation

All ingredients used in the formulation of adult diets for rearing *B. dorsalis* are shown in Table 2. The adult flies in this study were provided with unlimited access to two different yeast-based diets: a protein-rich dry diet and a liquid diet (Table 2). The yeast used in the diets not only contained protein but also provided essential B-complex vitamins, sodium chloride, lipids, and minerals. In addition, the casein used in dry adult diet was another source of protein. The source of carbohydrates was sugar. This formulation ensured that the flies received a well-rounded nutritional intake. Water was supplied using a cotton wick positioned in a conical flask filled with water.

**Table 2:** Formulations of adult diets for rearing *Bactrocera dorsalis.*

Ingredients	Source	Dry diet (per 1 kg)	Liquid diet (per 1 kg)
Yeast extract	Sigma-Aldrich^®^	250 g	-
Casein	Sigma-Aldrich^®^	250 g	-
Sugar	ACI Foods Ltd., Bangladesh	500 g	300 g
Baker’s yeast (Incubated in oven at 47 °C)	Angel Yeast Co., Ltd. China	-	100 g
Distilled water	Water distillation unit of laboratory	-	600 mL

Note: Incubate the entire mixture of adult liquid diet in an oven at 47 °C for up to 5 days.

**Fig 1:**
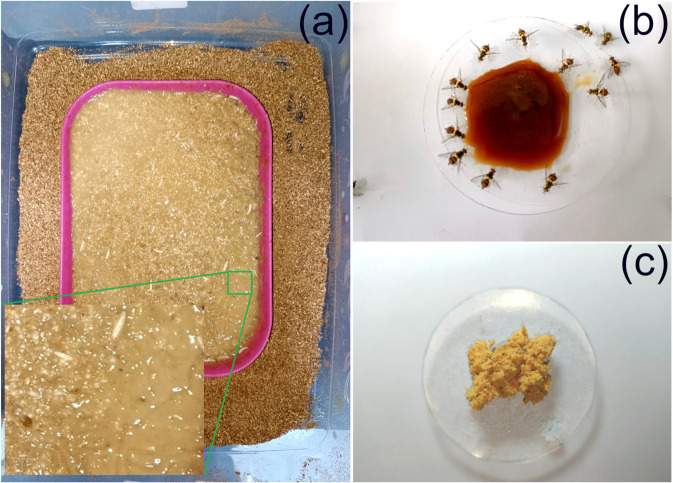
Artificial diets for mass rearing of *Bactrocera dorsalis*: (a) Gel-based meridic larval diet (b) Liquid adult diet (c) Dry adult diet.

The dry adult diet (Fig 1c) used in the experiment was prepared by directly mixing all the ingredients: casein, yeast extract, and sugar in the ratio of 1:1:2. In this dry adult diet formulation, casein has a water-binding capacity from the air due to its structure. This water binding capacity of casein allows it to hold a significant amount of water within its protein network. In addition to the dry adult diet, another long-lasting liquid diet was also provided (Fig 1b). This adult liquid diet consisted of distilled water, sugar, and baker’s yeast (*Saccharomyces cerevisiae*) (Table 2). The initial liquid diet mixture was incubated in an oven at 47 °C for five days until a thick syrup with high viscosity was formed. Both dry and liquid diets were offered to adults in separate watch glasses, allowing for ad-libitum consumption throughout the duration of the experiment.

### Nutrient analysis of adult and larval diets

Before using our new formulated adult and larval diets as rearing diets, a nutrient analysis was conducted using proximate analysis method at Food Safety and Quality Analysis Division of the Institute of Food and Radiation Biology, Atomic Energy Research Establishment, Bangladesh. The nutritional compositions of larval and adult diets were assessed using samples from three independent diet batches.

### Evaluation of larval development parameters

In this study, egg collection was performed using perforated plastic (0.5 mm diameter holes) container with egging stimulant banana puree. For mass rearing the larval stage and for determination of the relevant biological parameters, 0.5 mL eggs were seeded on larval rearing tray containing 333 mL larval diet. Up to pupation, the rearing trays were kept in laboratory conditions identical to those for adults. To measure the duration from egg seeding to the initiation of pupation, we maintained six replicates. The total number of pupae produced from 0.5 mL of eggs was counted. Three of these replicate samples were used to observe the average number of pupae produced. The quality parameters of the pupae (i.e., size, weight and adult emergence) were determined. Pupal weight was measured for 100 pupae, with 10 individuals randomly sampled from each of the 10 replicate groups. The adult emergence rate was determined by averaging the results of ten experiments. In each experiment, the number of pupae was in the range of 77 and 200, with a total of 1693 pupae. The sex ratio was determined by randomly sampling a minimum of 129 adults that emerged from each of the nine replicate batches of pupae (a total sample of 923 adults) to ensure a reliable estimate of the ratio. In addition, quality parameters of adult flies that emerged from these pupae were measured subsequently. To mitigate density-dependent negative effects on egg hatching rates as well as larval to pupal survival, a very low sample size (below 400) was maintained for the respective experiments [29]. Hatching rate was assessed using six replicate egg batches, each containing between 193 and 396 eggs (number of eggs: 193, 237, 295, 296, 332, 396 each corresponding batch), to provide a robust evaluation of hatching success. To measure the pupation rate, the total number of pupae derived from first instar larvae was calculated for each replicate. Four larval batches were used, with counts of 298, 335, 183, and 273 first instar larvae, respectively.

### Flight test

The assay was carried out according to FAO/IAEA/USDA quality control manual [21] with minor modifications. To evaluate the fitness of adult flies, we used a device made from a black PVC pipe. The pipe had a diameter of 9 cm and a height of 10 cm. The bottom of the pipe was sealed with a black paper-lined petri dish (9 cm in diameter). Prior to the flight test, the inner walls of the PVC pipe were dusted with odorless talcum powder to stop the flies from walking out. Flight performance was assessed in four replicate trials, each containing 100 pupae. A total of 100 pupae was placed at the base of the black PVC tube to evaluate the flight capability of adult fruit flies. After completing the process of adult emergence, fruit flies were categorized based on escape success. Fliers (those exiting the tube unaided), partially emerged individuals, deformed flies, and fully emerged non-fliers were counted to determine the flight ability. The percentage of flies that were able to fly was estimated using the following equation [30]:

$$Fliers_{\text{percentage}}=N_{\text{total}}-\left(\frac{N_{\text{not-emerged}}+N_{\text{partial}}+N_{\text{deformed}}+N_{\text{non-fliers}}}{N_{\text{total}}}\right)\times 100$$ (1)

Where,

$Fliers_{\text{percentage}}$ = Percentage of fliers

$N_{\text{total}}$ = Number of total pupae,

$N_{\text{not-emerged}}$ = Number of not emerged pupae

$N_{\text{partial}}$ = Number of partially emerged pupae

$N_{\text{deformed}}$ = Number of deformed pupae

$N_{\text{non-fliers}}$ = Number of non-fliers

### Morphometry

Different physical parameters of adult *B. dorsalis* including body length, wing length, thorax width, etc. were measured. In addition, pupal size and weight were also measured for *B. dorsalis* reared on larval and adult diet under laboratory conditions. To compare biological parameters, wild *B. dorsalis* adults were collected. The wild males were collected from the local population using methyl eugenol baited traps. On the other hand, the wild females were collected from six types of infested fruits (guava, wax apple, slow match tree, mango, and tropical almond). To minimize the influence of host fruit types, we selected six major types available for the wild population and maintained them in laboratory rearing conditions (27 ± 1 °C, 70 ± 5% relative humidity (RH), with a 14-h light:10-h dark photoperiod) until the emergence of adults. Moreover, the larval host is not the major determinant of adult body size in wild population of frugivorous Tephritidae fruit flies [9]. The available traps which are designed for population monitoring are not effective for capturing particular maturity aged females of *B. dorsalis* [31]. Due to this limitation, the ovary length of *B. dorsalis* wild population was not measured in our study. Morphometric measurements of body length and thorax width of laboratory reared adult fruit flies were measured with a sample size of 22 laboratory reared adults (11 males and 11 females). From the wild population, adult fruit flies body length and thorax width were measured with a sample size of 44 wild adult fruit flies (20 males and 24 females). Wing length measurements were collected from 44 wild adults (22 males and 22 females) and 32 laboratory-reared adults (16 males and 16 females).

Furthermore, morphometry of ovary and testes were also observed under a stereomicroscope (Leica S8 APO Stereo Microscope with Leica DMC2900 digital camera) where the necessary parameters like ovary length and testes length were measured. In our study, the length of the ovaries of laboratory-reared mature female fruit flies (sample size 11) was measured. Furthermore, testis lengths of mature fruit fly males were measured for both laboratory-reared (sample size of 11 males) and wild (sample size of 11 males) populations. First, sexually mature male and female flies (15 days old) were collected from adult rearing cages and the collected flies were killed in 70% ethanol before dissection. Next, the reproductive systems were extracted from the lower abdomen of adult flies. The reproductive systems were then carefully removed from the surrounding tissues following the dissection procedure described by Chou et al. [32]. Similar measurements were also taken for the collected wild male and female specimens, excluding the ovary of wild females.

### Survival analysis

Adult flies were placed in rearing cages immediately after their emergence to evaluate their longevity and survival. In one experiment, the flies were given unlimited access to the two diets (dry and liquid adult diets as mentioned in section: Adult diet formulation), where the two diets were provided in separate watch glasses (Fig 1b, 1c). For this study, the lifespan measurements were conducted with a sample size of 226 adult fruit flies (115 males and 111 females). In another experiment, longevity and survival under stress were assessed by providing only distilled water. The larval diet is considered the strongest determinant of starvation resistance [33]. To measure the effect of starvation on adult fruit flies that developed from gel based larval diet, the adults were given only water without any adult food. In this experiment, only water was supplied to prevent mortality due to desiccation. The lifespan of adult fruit flies given only water was measured with a sample size of 108 adult flies (50 males and 58 females).

For adult survival analysis, both male and female data were calculated separately as their longevity was different. In this study, we recorded the mortality rates on a daily basis. The survival curves of both males and females those kept under similar conditions were compared with each other.

### Statistical analysis

For all statistical analyses, OriginPro^®^ software was used. Descriptive statistics were used to measure the mean and standard deviation (SD) of several key biological parameters (pupal weight, hatching rate, number of pupae produced, pupation rate, duration for commencement of pupation, adult emergence rate, flying rate, morphometric characteristics and lifespans) of *B. dorsalis*. Two-tailed t-test was performed to compare different groups. We used Tukey box plots to show the comparison of biological parameters (body length, testis size and longevity) of *B. dorsalis*. Pearson’s correlation coefficient was used to determine the relationship between the testis length and body length. Two-tailed t-test was conducted to determine gender-dependent variation in longevity when adults were provided with either only water or a complete diet. In this study, survival analyses were performed using two different widely used survival analysis methods (non-parametric Kaplan–Meier estimator and Weibull parametric model) by biologists. In addition to that the log-rank (Mantel-Cox) test was used to compare the survival curves between sexes for both the full adult diet supplement group and the water-only group. A significance level of *α* = 0.05 was used for all statistical tests.

The Kaplan–Meier estimator [34,35] is a non-parametric statistic used to estimate the survival function *S*(*t*). It calculates the probability of an event (like death or failure) not occurring over time, without assuming any specific distribution for survival times even with incomplete observations (censored data). The estimator is defined by the product-limit formula:

$$S(t)=\prod_{t_{i}\leq t}\left(1-\frac{d_{i}}{n_{i}}\right)$$ (2)

Here, *t*_*i*_ denotes the time of each distinct event (e.g., death or failure), d_i_ is the number of events at time *t*_*i*_, and n_i_ is the number of individuals at risk (i.e., still alive and in the study) just before *t*_*i*_. While representing graphically, the survival function *S*(*t*) is a step function with abrupt drops at each event time, visually represents survival probability. It is widely used for studying time-to-event data like insect mortality under experimental conditions. This method accounts for censored observations (e.g., insects still alive at the end of a study) and is favored for its flexibility in handling incomplete data. Furthermore, this method can be used as a benchmark for evaluating parametric model fits for survival analysis.

The Weibull parametric model [35,36] is a widely used and adaptable method in survival analysis for examining time-to-event data. Its versatility comes from its ability to model different types of risk patterns over time. It can represent the failure rate of a population in several ways: as a decreasing, constant, or increasing rate over time. The Weibull distribution is characterized by its scale parameter (λ>0), which affects the timeline of events and shape parameter (*k* > 0), which dictates the nature of the hazard rate.

The hazard function, which describes the instantaneous risk of an event at time *t*, is defined as:

$$h(t)=\lambda \;k\;(\lambda \;t{)}^{\;k-1}$$ (3)

Consequently, the survival function, representing the probability of an individual surviving past time *t*, is calculated with the formula:

$$S(t)=\exp\;[-(\lambda \;t{)}^{\;k}]$$ (4)

A key advantage of the Weibull model is its ability to represent various hazard scenarios: an increasing risk over time (when (*k* > 1), a decreasing risk (when (*k* < 1), or a constant risk (when (*k* = 1), which simplifies to the exponential model). Because it is a parametric model, it provides a clear way to estimate the impact of other variables, making it a highly versatile tool for survival analysis.

By employing both the Kaplan-Meier estimator and the Weibull parametric model in survival analysis, we can gain a comprehensive perspective on survival dynamics. The Kaplan-Meier estimator offers a reliable, assumption-free depiction of observed survival, while the Weibull model provides a model-based interpretation, enabling extrapolation and the estimation of key parameters. Conducting analyses with both methods enhances the credibility of our results, allowing for a direct comparison between empirical data and model projections. Strong agreement between the Weibull model and Kaplan-Meier estimates within the observed time frame leads to greater confidence in long-term predictions and the overall validity of our survival analysis [37].

## Results and discussion

### Nutrient analysis of larval and adult diets

Analyses of nutrient composition of gel-based larval diet and adult diets (dry and liquid) used for *B. dorsalis* rearing (Fig 1) revealed actual ratio of moisture, protein, fat, carbohydrate, and ash content. Table 3 shows the mean values of these nutrient contents in percentage with standard deviation (SD).

**Table 3:** Nutrient contents of larval and adult diets used for rearing *Bactrocera dorsalis.*

Nutrient	Gel-based larval diet (Mean ± SD)%	Dry adult diet (Mean ± SD)%	Liquid adult diet (Mean ± SD)%
Moisture	72.22 ± 0.39	8.67 ± 0.33	20.78 ± 0.69
Protein	5.83 ± 0.20	35.82 ± 0.73	7.99 ± 0.10
Fat	2.48 ± 0.01	5.52 ± 0.09	1.04 ± 0.01
Carbohydrate	18.73 ± 0.15	47.48 ± 0.45	69.41 ± 0.56
Ash	0.73 ± 0.03	2.52 ± 0.03	0.78 ± 0.05

Note: The analysis of the larval diet was conducted after gelation process.

Once the diets were carefully formulated, we took precise measurements of the pH levels prior to offering them to the fruit flies. The initial pH measurements, as shown in Table 4, revealed that all the formulated gel-based larval diet and adult diets were slightly to moderately acidic. The pH level of our formulated gel-based larval diet was 4.66 ± 0.006. The pH level of a similar gel-based larval diet reported in 2019 by Mahfuza Khan et al. was in the range of 3.5 to 4 [15]. The different composition ratios of the ingredients used in our larval diet formulation resulted in higher pH level. Since our diet formulation does not include bacteria as probiotics, it allows for a reduction in production costs, making it a more economical option without compromising on quality. Moreover, maintaining slightly to moderately acidic pH levels in our formulated gel-based larval diet and adult diets can effectively suppress the growth of harmful microbes, thereby promoting better health for fruit flies.

**Table 4:** Initial acidity or alkalinity of larval and adult diets used for rearing *Bactrocera dorsalis.*

Category of diet	Acidity or alkalinity (pH) (Mean ± SD)
Gel-based larval diet	4.66 ± 0.006
Dry adult diet	6.56 ± 0.01
Liquid adult diet	5.17 ± 0.01

Note: The pH analysis was conducted after diet preparation was completed.

### Influence on egg hatching rate and pupation

The egg hatching rate observed for the gel-based larval diet was 90.98 ± 3.53%, which is very close with the value 90.92 ± 0.9% reported in 2009 by Chang for the larval liquid diet [24]. The hatching rate of *B. dorsalis* in an artificial diet (wheat bran media) reported by Suhana et al. was 84.3%, which is lower compared to our larval diet [38]. According to previous reports by Chang [24] and Pieterse et al. [39], the observed hatching rate in this study indicates that our formulated diet is suitable for mass rearing. The pupation rate observed was 94.49 ± 2.21%, indicating that most first instar larvae successfully developed into pupae with the provided larval diet conditions. In our pupal production study, 0.5 mL eggs were seeded into 333 mL gel-based larval diet (as described in materials and methods). Mean number of eggs in the 0.1 mL volume was 863.67 ± 56.22. From three replicate samples, the observed average number of pupae was 3332.67 ± 326.79. The average time from egg seeding to the commencement of pupation was 7.33 ± 0.52 days.

### Influence on pupal weight

Different quality parameters (including pupal size and weight) of *B. dorsalis* were also observed for the agar-based larval diet. The average weight of the pupae was 15.18 ± 0.48 mg, as shown in Table 5. According to the IAEA’s mass rearing quality control guidelines for tephritid flies [21], for SIT programs the required minimum pupal weight is 12.30 mg, and acceptable mean pupal weight is 12.90 mg. The pupae reared on our diet exceeded the required average pupal weight by about 17.67% and minimum pupal weight by about 19.51%.

**Table 5:** Quality parameters of larval rearing diet for *Bactrocera dorsalis.*

Quality parameters of larval rearing diet	Mean ± SD
Pupal weight (mg)	15.18 ± 0.48
Hatching rate (%)	90.98 ± 3.53
Number of pupae produced from 0.5 mL eggs	3332.67 ± 326.79
Pupation rate (%)	94.4925 ± 2.21
Egg seeding to commencement of pupation (days)	7.33 ± 0.52
Adult emergence rate from pupae (%)	96.88 ± 1.56
Flying rate (%)	86.75 ± 2.06

### Influence on adult emergence rate

In our experiments, we observed an adult emergence rate of 96.88 ± 1.56% (Table 5), which exceeds the International Atomic Energy Agency (IAEA) standards for mass rearing [21]. This adult emergence rate (pupa-to-adult fly development) exceeded both the minimum threshold of 82% as well as the acceptable threshold of 90%. The adult emergence rate of *B. dorsalis* reared on our diet exceeded the required rate by about 6.88 ± 1.56%. The observed rate is also comparable to the 97.33% eclosion rate for *B. dorsalis* reported by Chen et al. [40]. The analysis revealed a male-to-female ratio of 1.10:1, indicating a slight male dominance (see Table 6). These results demonstrate that our formulated diet meets the necessary quality control standards for mass rearing of *B. dorsalis* suggested by IAEA.

**Table 6:** Morphometric traits of adult *Bactrocera dorsalis.*

Origin of flies	Morphometric characteristics	Mean ± SD
**Laboratory reared male**	Body length (mm)	6.77 ± 0.20
	Thorax width (mm)	2.45 ± 0.16
	Wing length (mm)	6.15 ± 0.12
	Testes length (mm)	1.21 ± 0.17
**Laboratory reared female**	Body length (mm)	7.05 ± 0.34
	Thorax width (mm)	2.35 ± 0.19
	Wing length (mm)	6.34 ± 0.12
	Ovipositor length (mm)	4.08 ± 0.49
	Ovary length (mm)	2.20 ± 0.29
**Wild male**	Body length (mm)	6.44 ± 0.45
	Thorax width (mm)	2.21 ± 0.19
	Wing length (mm)	6.18 ± 0.36
	Testes length (mm)	1.19 ± 0.17
**Wild female**	Body length (mm)	6.01 ± 0.51
	Thorax width (mm)	2.04 ± 0.21
	Wing length (mm)	5.65 ± 0.52
**Laboratory reared adult sex ratio**	Male to female ratio	1.10:1

### Influence on body and wing size

**Fig 2:**
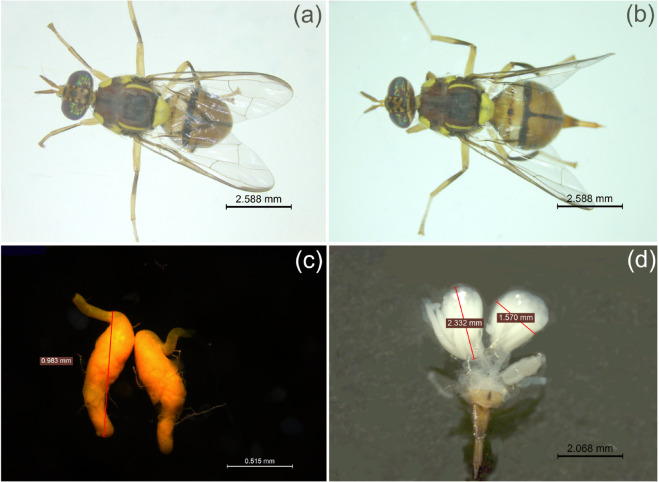
Morphological details of *Bactrocera dorsalis*: (a) Adult male (b) Adult female (c) Testes and (d) Ovaries.

**Fig 3:**
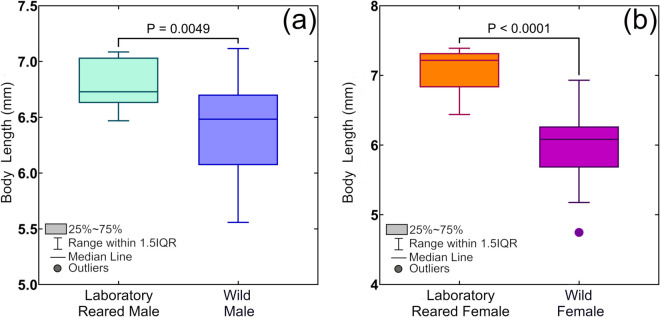
Comparison of body length of *Bactrocera dorsalis* for laboratory reared and local wild population using Tukey box plots: (a) Adult males and (b) Adult females.

Morphological details of *B. dorsalis* are shown in Fig 2. Different morphometric characteristics of the adults captured from wild environment as well as our laboratory reared adults fed with our formulated diet were measured (see Table 6). The Tukey box plots in Fig 3 show comparison of body length of laboratory reared and local wild *B. dorsalis* population. It was observed that laboratory-reared *B. dorsalis* adults, both male and female, were significantly larger than their wild counterparts. Specifically, laboratory-reared females were significantly larger than wild females (p < 0.0001), and laboratory-reared males were significantly larger than wild males (p = 0.0225).

In addition, while comparing wing size, we observed that adult male flies had a wing size of 6.15 ± 0.12 mm, whereas females had a slightly larger wing size of 6.34 ± 0.12 mm (see Table 6). Previously, 6.00 mm wing size of *B. dorsalis* was reported by Drew et al. [41]. The nutritional content of our formulated diet resulted in larger wings and body size, which is crucial for successful mating and population growth in laboratory conditions.

Previous research indicated that females, irrespective of their size, exhibited a preference for mating with larger males, suggesting potential advantages associated with choosing larger mates [42]. Furthermore, larger males have been proven to exhibit greater pre- and post-copulatory reproductive success [43]. These findings suggest that the current larval-rearing diet is adequate for promoting optimal growth and overall fitness of adult flies.

### Influence on flight ability

The ability of adult *B. dorsalis* to fly is crucial for the survival and reproductive success of their population. In our study, flight ability of adults was 86.75 ± 2.06%, as shown in Table 5. The IAEA mass rearing manual states that *B. dorsalis* adult flies that can be employed in SIT programs should have at least 75% flight ability, whereas the ideal level of flight ability is 83% [21]. The measured flight ability in our study meets the standard requirements for mass rearing.

### Influence on gonad development

Adult tephritid fruit flies require dietary protein for sexual maturation and gonad development [44,45]. Therefore, the adult flies were provided with unlimited access to two different yeast-based diets: a protein-rich dry diet and a liquid diet. In this study, we used the ovary and testes development (see Table 6) as the measurement parameters to evaluate the adult-rearing diet. Ovary length are reliable indicators to determine the physiological states (i.e., nutritional state) of *B. dorsalis* [32]. Therefore, ovary length is sufficient for comparison of growth rate and effects of diet.

We observed that the mean ovary length of 15-day-old gravid females was 2.20 ± 0.29 mm (see Table 6), which is larger than the previously reported ovary length of 1.8 ± 0.07 mm [32].

**Fig 4:**
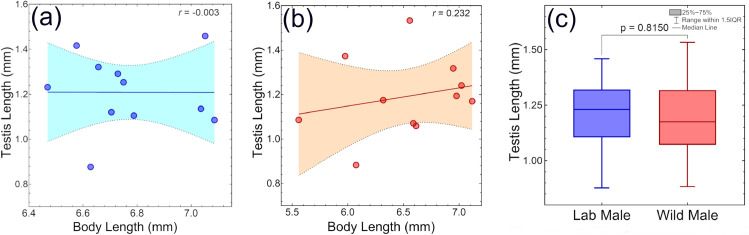
Correlation between male body size and testis size of *Bactrocera dorsalis*: (a) Laboratory-reared *B. dorsalis* (b) Wild *B. dorsalis*. The color shadows represent 95% confidence interval. (c) Comparison of testis size between laboratory-reared and wild male *B. dorsalis*.

Furthermore, we evaluated the correlation between male body size and testis size in both laboratory-reared and wild *B. dorsalis*. Fig 4b shows a simple linear regression analysis which demonstrates a weak positive correlation between body size and testes size in wild males (r = 0.232, p = 0.4919). A similar size difference in testes between large and small males has also been reported for walnut flies (*Rhagoletis juglandis*) [46]. In the research work by Carsten-Conner et al. [46], it was indicated that larval environment significantly influences testes size in male flies. However, this relationship was not observed in laboratory-reared males (r = -0.003, p = 0.9933) (see Fig 4a). This difference may be attributed to the more uniform growth observed in lab-reared males, with body lengths ranging from 6.47 mm to 7.09 mm. In contrast, wild males exhibited a wider range of body lengths (5.56 mm to 7.12 mm) (see Fig 3a), possibly reflecting the influence of variable natural resources and environmental conditions on their growth and development. The small variation in body size of males in laboratory settings could explain the absence of a considerable impact on testis size in laboratory-reared males. The results in this study were consistent with the prediction that body size of males does not always reflect the testes size in laboratory condition on *Drosophila melanogaster* [43]. Furthermore, while comparing testes size of wild and lab-reared male, we found that the mean testes size for wild males was 1.19 ± 0.18 mm (see Table 6), whereas the lab-reared males had a mean testes size of 1.208 ± 0.1664 mm. An unpaired t-test (Fig 4c) revealed that there is no statistically significant difference in testes size between the two groups (t = 0.2371, df = 20, p = 0.8150). However, previous research showed that larger male Tephritidae transfer much greater quantities of spermatozoa during mating [47]. This competitive advantage can maximize reproductive success of laboratory reared larger males.

### Influence on longevity and survival analysis

**Fig 5:**
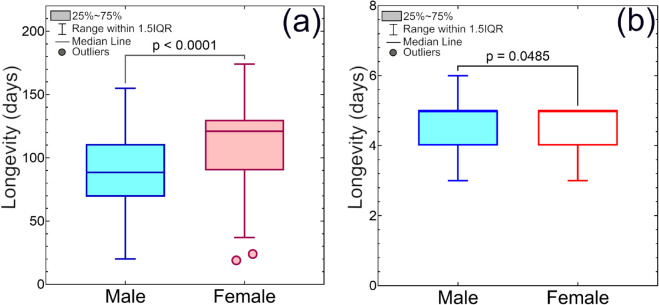
Comparison of longevity of adult male and female *Bactrocera dorsalis* using Tukey box plots: (a) Longevity of fed with carbohydrate and protein-rich food supply (b) Only distilled water supply. The median line coincides with the upper level of the box in Fig (b).

The lifespan of adult fruit flies was assessed for individuals maintained solely on water and for those provided full adult diet supplement. Fig 5 shows a change in longevity between male and female *B. dorsalis* under different dietary conditions. Females exhibited greater vulnerability to dietary restrictions, particularly protein and carbohydrate deprivation, as compared to their male counterparts.

**Fig 6:**
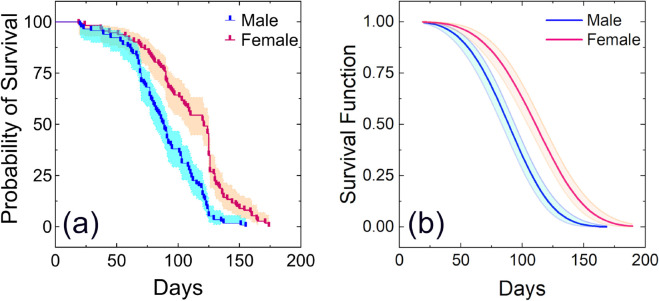
Survival curves of males (n = 116) and females (n = 112) of adult *Bactrocera dorsalis* fed with a carbohydrate and protein-rich food supply: (a) Survival curves using Kaplan–Meier estimator (b) Survival curves using Weibull parametric model. The color shadows over the survival curves, shown in Figs (a) and (b), represent the 95% confidence interval.

**Fig 7:**
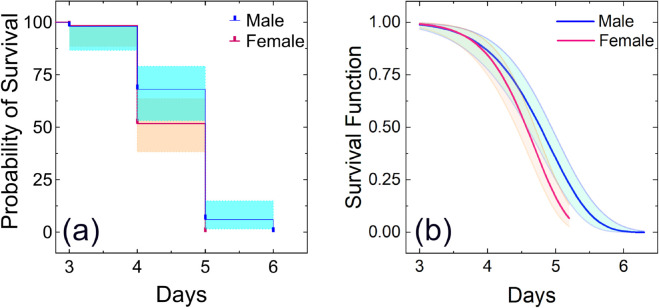
Survival curves of males (n = 50) and females (n = 58) of adult *Bactrocera dorsalis* fed with only distilled water: (a) Survival curves using Kaplan–Meier estimator (b) Survival curves using Weibull parametric model. The color shadows over the survival curves, shown in Figs (a) and (b), represent the 95% confidence interval.

Furthermore, the survival analyses (Figs 6 and 7), using non-parametric Kaplan–Meier estimator and Weibull parametric model, were conducted separately for each sex for observing differences in longevity. The survival curves obtained from the survival analyses revealed an age-dependent mortality pattern, characterized by an initial increase and followed by a plateau (Figs 6 and 7).

A significant difference in mean longevity was observed between adults of two groups: one group was only provided with distilled water, whereas the other group was provided with access to a complete diet. These findings point out the critical influence of diet composition, specifically protein and carbohydrate availability, on adult *B. dorsalis* survival. Furthermore, significant differences in sex-specific longevity patterns were observed under laboratory rearing conditions with access to protein and carbohydrate-rich food, where females exhibited significantly longer lifespans than males, as shown in Fig 6a, 6b. Similar findings were also reported by Jaleel et al. and Pieterse et al. [39,48].

Welma Pieterse et al. reported that the male and female lifespans of *B. dorsalis* were 42.3 ± 25.3 days and 40.03 ± 25.7 days, respectively, when fed a 3:1 mixture of sugar and yeast [39]. However, survival analyses in this study showed significantly longer lifespans: 110.26 ± 33.11 days for females and 88.75 ± 28.21 days for males when provided with two types of adult food. These lifespans also exceed those reported by Chen et al. across all the tested diets [49].

The log-rank test indicated a statistically significant difference in survival curves between sexes for both the full adult diet supplement group (p < 0.0001) and the water-only group (p = 0.0368). The survival analyses, as shown in Fig 7a, 7b, suggest that adults may survive only about 3 to 6 days with only water without any other food sources. In this case, the average longevity of females was approximately 4.50 ± 0.54 days, while the males had a slightly longer average longevity of about 4.72 ± 0.61 days. This survival period is enough for adult flies to disperse over long distances in search of food within the release area.

Overall, this study showed that the survival of *B. dorsalis* varied significantly depending on their food sources and sex. Similar findings were also reported by previous researchers [49]. These results demonstrate that *B. dorsalis* developed well on our formulated larval and adult diets, suggesting significant potential for the use of these formulated diets in mass laboratory-rearing of *B. dorsalis* larvae and adults for Sterile Insect Technique (SIT) program.

## Conclusions

In this study, the effectiveness of our new formulated agar-based larval and yeast-based adult diets for lab-reared *B. dorsalis* was investigated. Statistical analyses including Tukey tests and survival analyses were performed for understanding the efficacy of our formulated diets. Our gel-based larval diet ensured required standard pupal weight and required adult emergence rate recommended by FAO/IAEA/USDA quality control manual for mass rearing of Tephritidae fruit fly in laboratory. Compared to wild flies, laboratory-reared adults exhibited a notably larger body size. Additionally, our gel-based larval diet formulation reduces waste production by eliminating the bulking agents typically required in larval diets. In addition to larval diet, our protein-based adult diets also exhibited improved parameters related to adult rearing. Adult flies that were fed yeast-based diets produced eggs with a high hatching rate when the eggs were seeded onto the gel-based larval diet. Lab-reared flies using our formulated diet showed superior flight ability, exceeding required mass rearing standards. In addition, it was observed that the ovary of lab-reared female flies was larger in length as compared to previous reports, whereas the size of lab-reared male testes remained comparable to wild flies. Furthermore, this study showed that depending on food sources and sex, the survival of *B. dorsalis* varied significantly. While the adults survived only a short period on a water diet without food supply, our formulated complete adult diets showed significantly increased longevity. Importantly, the formulated diets employed for the rearing of larvae and adults in this study adhere to all the minimum quality standards outlined in the FAO/IAEA/USDA quality control manual. Moreover, our formulated diets also fulfilled established protocols requirement for mass rearing for the release of sterile males of *B. dorsalis*. The integration of our new formulated diets into the mass rearing process of *B. dorsalis* in a laboratory setting has the potential to significantly improve the production of larger and healthier flies on a large scale, exhibiting improved flight capabilities and reproductive parameters.
